# The antibody response to SARS-CoV-2 infection persists over at least 8 months in symptomatic patients

**DOI:** 10.1038/s43856-021-00032-0

**Published:** 2021-09-17

**Authors:** Riccardo Levi, Leonardo Ubaldi, Chiara Pozzi, Giovanni Angelotti, Maria Teresa Sandri, Elena Azzolini, Michela Salvatici, Victor Savevski, Alberto Mantovani, Maria Rescigno

**Affiliations:** 1 Department of Biomedical Sciences, Humanitas University, Via Rita Levi Montalcini 4, 20072 Pieve Emanuele Milano, Italy; 2 IRCCS Humanitas Research Hospital, Via Manzoni 56, 20089 Rozzano Milano, Italy; 3 The William Harvey Research Institute, Queen Mary University of London, London, UK

**Keywords:** Viral infection, Epidemiology, Computational biology and bioinformatics

## Abstract

**Background:**

Persistence of antibodies to SARS-CoV-2 viral infection may depend on several factors and may be related to the severity of disease or to the different symptoms.

**Methods:**

We evaluated the antibody response to SARS-CoV-2 in personnel from 9 healthcare facilities and an international medical school and its association with individuals’ characteristics and COVID-19 symptoms in an observational cohort study. We enrolled 4735 subjects (corresponding to 80% of all personnel) for three time points over a period of 8–10 months. For each participant, we determined the rate of antibody increase or decrease over time in relation to 93 features analyzed in univariate and multivariate analyses through a machine learning approach.

**Results:**

Here we show in individuals positive for IgG (≥12 AU/mL) at the beginning of the study an increase [*p* *=* *0*.*0002*] in antibody response in paucisymptomatic or symptomatic subjects, particularly with loss of taste or smell (anosmia/dysgeusia: OR 2.75, 95% CI 1.753 – 4.301), in a multivariate logistic regression analysis in the first three months. The antibody response persists for at least 8–10 months.

**Conclusions:**

SARS-CoV-2 infection induces a long lasting antibody response that increases in the first months, particularly in individuals with anosmia/dysgeusia. This may be linked to the lingering of SARS-CoV-2 in the olfactory bulb.

## Abstract (web summary)

Levi and Ubaldi et al. evaluate SARS-CoV-2 seroprevalence in a cohort of 4735 healthcare workers in northern Italy. In seropositive individuals, they show that antibodies are maintained over a period of 8 to 10 months and associate changes in antibody levels over this period with symptoms and specific subgroups of participants.

## Plain language summary

SARS-CoV-2 infection activates the body’s immune system to fight off infection. This immune response results in the production of proteins in the blood that target the virus called antibodies. The extent and duration of this antibody response may be associated with the type of symptoms the infected person is experiencing. Here, we analyzed SARS-CoV-2 antibody levels in individuals with asymptomatic, mild symptomatic (paucisymptomatic) and symptomatic disease in relation to the type of symptoms. We find that the antibody response is higher in people with symptoms and increases in the first three months, particularly in individuals with loss of smell or taste. In all people with SARS-CoV-2 antibodies at the start of the study, levels in the blood last for at least 8–10 months. Hence, SARS-CoV-2 infection is characterized by a long lasting antibody response which may protect from subsequent infections.

## Introduction

It is becoming clear that the antibody response to SARS-CoV-2 can last at least 6 months in symptomatic patients^1^, but it seems to decline in asymptomatics^2^, including children^3,4^. Similarly, a reduction of antibody response in asymptomatic individuals was shown in a study with a fewer number of individuals (*n* = 37)^5^. In another study with a small number of subjects (*n* = 68), both IgG and IgA were shown to remain remarkably high for a 4 month period of time^6^. The antibody response in COVID-19 patients is associated with the establishment of a memory B cell response which is higher at 6 months^1^, however, it is not clear whether there are features that correlate with this sustained B cell response. In addition, the antibody response to the spike protein might last for at least 7 months, while that to nucleocapsid wanes over time^7^ and this correlates with a reduction in the IFN-γ producing CD8 + T cell response to the nucleocapsid^8^. The quality of an antibody response is fundamental to avoid long-term effects of COVID-19, indeed individuals with low or waning antibody response are more subject to long-COVID^9^. Hence, it is imperative to investigate the association between the antibody response, its duration, and the type of symptoms. We previously showed that an anti-SARS-CoV-2 serological analysis allowed us to follow the diffusion of the virus within healthcare facilities in areas differently hit by the virus^10^. At 8–10 months of distance, we analyzed the duration of this antibody response and evaluated whether there were features correlating with maintenance, reduction, or increase of the antibody response. We found that SARS-CoV-2 antibody levels increase in symptomatic subjects (particularly in individuals with anosmia/dysgeusia) in the first 3 months of the study. Moreover, this antibody response lasts for at least 8–10 months in all people with SARS-CoV-2 antibodies at the start of the study.

## Methods

### Study design

This observational cohort study aimed at determining the anti-SARS-CoV-2 IgG plasma levels in nearly 4000 employees of nine healthcare facilities and an international medical school located in northern Italy. It has been approved by the institutional review board of Istituto Clinico Humanitas (ICH) for all participating institutes (ID:1374 IgG COVID-19 Humanitas and registered on clinicaltrial.gov NCT04387929). We have adhered to the STROBE reporting guidelines for observational studies. The serological determination was offered to all the employees of the involved sites and the anticipated refusal rate was assumed to be 10–15%. The overall IgG positivity was assumed to be around 10–15%. The primary endpoint was the number of test positive subjects. Given the study size, the study was able to estimate the overall positivity with a width of 95% confidence interval equal to 2% and the positivity for subgroups of at least 200 patients with a width of 95% confidence interval equal to 9%. No power analysis was performed to calculate the sample size. No randomization was performed. Accrual was on a voluntary basis (individuals aged ≥18 years; Humanitas group employees): it started on April 28th 2020 and more than 80% of personnel participated (*n* = 4735). The study foresees four blood collections every 3/4 months. Ten different centers participate: ICH, Rozzano (MI); Humanitas Gavazzeni, Bergamo; Humanitas Castelli, Bergamo; Humanitas Mater Domini (HMD), Castellanza (VA); Humanitas Medical Center (HMC), Varese; Humanitas University, Pieve Emanuele (MI); Humanitas San Pio X, Milano; Humanitas Cellini, Torino; Humanitas Gradenigo, Torino; Clinica Fornaca, Torino. This study was conducted following the Helsinki principles of good clinical practice and all participants signed informed consent and filled an anamnestic questionnaire before blood collection. In order to be tested, subjects had to fill in the questionnaire. Only after filling the questionnaire in its entirety, would the individuals be scheduled for blood sampling. We analyzed 93 features (72 categorical and 17 numerical and four temporal) including, age, sex, location, professional role, the time between sample collections, COVID-19 symptoms (fever, sore throat, cough, muscle pain, asthenia, anosmia/dysgeusia (loss of smell and taste), gastrointestinal symptoms, conjunctivitis, dyspnea, chest pain, tachycardia, and pneumonia), home exits and smart-working, comorbidities (diabetes, asthma, neoplasia, autoimmunity, cardiovascular disorders, and hepatic disorders). We considered “asymptomatics” subjects without any symptoms; “paucisymptomatics” individuals that developed 1 or 2 symptoms; “symptomatics” individuals with three or more than three symptoms. None of the participants were enrolled at the time of symptoms. Thus, when the serological test was performed, they were either asymptomatics or the symptoms had disappeared. After excluding for employees that became positive for SARS-CoV-2 IgG (*n* = 2) during the observation period and those that dropped from phase 1 or for which we were missing at least two features, we analyzed 4534 participants (4.25% drop out) that participated to both phase 1 (April–May 2020) and phase 2 (July–August 2020). Among these, 499 subjects participated also to phase 3 (November–December 2020).

### IgG measure

For the determination of IgG anti-SARS-CoV-2, the Liaison SARS-CoV-2 S1/S2 IgG assay (DiaSorin, Saluggia (VC), Italy) was used^11^. The method is an indirect chemiluminescence immunoassay for the determination of anti-S1 and anti-S2 specific antibodies. According to kit manufacturer, the test discriminates among negative (<15 AU/mL; with 3.8 as the limit of IgG detection) and positive (≥15 AU/mL) subjects. We considered positive subjects with IgG plasma levels ≥12 AU/mL rather than those with IgG ≥15 AU/mL, as suggested by the test manufacturer, based on our previous publication showing that these two groups behaved very similarly^10^. In addition, we considered also individuals with IgG comprised between 3.8 and 12 AU/mL (which we called IgG med: 3.8 < IgG <12 AU/mL). Consistency and reproducibility of the antibody test in samples collected in the two time points was confirmed for a limited number of individuals (*n* = 50) displaying different degrees of IgG positivity. The LIAISON assay’s performance in comparison to a microneutralization assay is shown in Bonelli et al^11^. The LIAISON serological S1/S2 assay can distinguish between neutralization positive and negative samples at cut-offs near 15 AU/mL, and additionally, the data indicate that 92% of the samples with >80 AU/mL had neutralization titers ≥1:80, while 87% of samples with >80 AU/mL had neutralization titers ≥1:160.

As the samples were analyzed in separate batches, we compared the test accuracy on 21 samples from the phase 1 with the detection kits of phase 1 and phase 2 and demonstrated that the tested IgG were almost over-imposable (Supplementary Fig. 3).

### Statistical analysis and model

We first cleared the dataset by eliminating data from all of those subjects that did not develop an IgG response over time (IgG ≤3.8 at the beginning and at the end of the examination) (*n* = 2981). We then analyzed the rate of antibody response defined as:

$${{{{{\mathrm{RATE}}}}}}=\frac{{{{{{\mathrm{IgG}}}}}}\,{{{{{\mathrm{phase}}}}}}\,2-{{{{{\mathrm{IgG}}}}}}\,{{{{{\mathrm{phase}}}}}}\,1}{\Delta {{{{{\mathrm{days}}}}}}}=[{{{{{\mathrm{AU}}}}}}/{{{{{\mathrm{mL}}}}}}* {{{{{\mathrm{day}}}}}}]$$

Positive rates mean increased antibody response, while negative rates indicate reduction of antibody response between the two analyzed time points.

For statistical analysis, we performed both a univariate and multivariate analysis. We applied Wilcoxon–Mann–Whitney statistical nonparametric test to compare the antibody rate distribution between classes of subjects (Tables 1 and 2).

**Table 1:** Demographic distribution of antibody rates.

		counts	min	25 perc	50 perc	75 perc	max	mean	St.dev.	*p* value^a^
Sex	F	1105	−3.39	−0.04	−0.02	0.02	16.32	0.07	0.61	0.0139
	M	448	−0.88	−0.04	−0.02	0	10.37	0.03	0.53	
Age	21–30	300	−3.39	−0.04	−0.02	0.01	4.51	0.03	0.41	0.2644
	31–40	365	−0.92	−0.04	−0.02	0.01	2.26	0.02	0.21	0.2302
	41–50	455	−2.49	−0.04	−0.02	0.03	3.11	0.06	0.37	0.1083
	51–60	309	−0.88	−0.04	−0.01	0.02	16.32	0.1	0.96	0.1442
	60+	124	−0.68	−0.04	−0.02	−0.01	10.37	0.12	0.99	0.0586
BMI^b^	18.5≤ BMI <25	940	−0.92	−0.04	−0.02	0.01	10.37	0.03	0.41	0.3821
	BMI ≥30	106	−3.39	−0.05	−0.02	0.14	16.32	0.23	1.69	0.2182
	25≤ BMI <30	347	−0.57	−0.04	−0.02	0.01	3.11	0.05	0.29	0.3933
	BMI <18.5	73	−0.22	−0.04	−0.02	0.02	1.27	0.05	0.26	0.3959
IgG class phase 1	IgG ≥12	613	−3.39	−0.08	0.02	0.23	16.32	0.18	0.92	2.1 E-10
	IgG ≤3.8	74	0.01	0.01	0.02	0.03	0.08	0.02	0.01	1.5 E-15
	3.8 < IgG <12	866	−0.13	−0.03	−0.02	−0.01	0.83	−0.02	0.04	8.0 E-22
Role	Other^c^	200	−0.57	−0.04	−0.02	−0.01	16.32	0.09	1.17	0.0116
	Anesthesiologist	19	−0.83	−0.04	−0.02	0.01	0.09	−0.05	0.2	0.4305^d^
	Biologist	18	−0.18	−0.05	−0.02	−0.02	0.03	−0.04	0.05	0.0978^d^
	Surgeon	67	−0.88	−0.05	−0.02	0.07	0.61	0.02	0.21	0.2653
	Physiotherapist	21	−0.12	−0.04	−0.02	0.01	0.35	0.01	0.1	0.4204
	Nurse	398	−3.39	−0.04	−0.02	0.04	3.11	0.08	0.41	0.0581
	Physician	210	−0.92	−0.04	−0.01	0.02	10.37	0.08	0.75	0.0804
	Healthcare partner operator	149	−2.49	−0.03	−0.01	0.17	1.55	0.1	0.38	0.0009
	Front office (PARC)	108	−0.32	−0.04	−0.02	0.01	2.55	0.03	0.27	0.2892
	Researcher	50	−1.5	−0.03	−0.02	−0.01	4.51	0.06	0.68	0.1514
	Cleaning service	29	−0.65	−0.03	−0.02	0.03	1.72	0.09	0.41	0.2414
	Transport service	14	−0.39	−0.04	−0.01	0.02	2.22	0.13	0.62	0.4359^d^
	Staff	188	−0.4	−0.04	−0.02	−0.01	0.98	0	0.15	0.0026
	Student	20	−0.19	−0.03	−0.02	−0.01	0.21	−0.02	0.08	0.3705^d^
	Laboratory technician	31	−0.17	−0.03	−0.01	−0.01	0.51	0.02	0.15	0.3243
	Radiology technician	31	−0.18	−0.03	−0.02	0.01	1.01	0.04	0.22	0.4002
Site	Other^c^	21	−0.19	−0.04	−0.02	−0.01	2.22	0.08	0.49	0.2080
	Casa di Cura Cellini	51	−0.17	−0.05	−0.03	−0.01	1.27	0.01	0.22	0.0111
	Clinica Fornaca di Sessant	47	−0.4	−0.05	−0.03	0	0.53	0	0.15	0.0833
	Humanitas Castelli	87	−0.28	−0.04	0.02	0.21	3.11	0.24	0.62	4.7 E-05
	Humanitas Gavazzeni	313	−0.88	−0.04	−0.01	0.15	10.37	0.13	0.68	0.0001
	Humanitas Gradenigo	109	−2.49	−0.04	−0.02	−0.01	0.67	−0.01	0.27	0.0586
	Humanitas Mater Domini	105	−0.17	−0.03	−0.02	−0.01	0.63	0	0.1	0.3412
	Humanitas Medical Care	23	−0.19	−0.04	−0.02	−0.01	0.02	−0.03	0.04	0.0969
	Humanitas Rozzano	667	−3.39	−0.04	−0.02	0	16.32	0.04	0.7	0.0338
	Humanitas San Pio X	98	−0.68	−0.03	−0.02	0	2.39	0.04	0.29	0.2968
	Humanitas University	32	−0.2	−0.04	−0.02	−0.01	0.19	−0.03	0.08	0.0590

^a^Wilcoxon–Mann–Whitney test.

^b^Some subjects did not indicate their BMI.

^c^Refers to volunteers and other professionals that operate in several structures.

^d^Minority class is less or equal to 20 (Wilcoxon–Mann–Whitney test is not reliable).

**Table 2:** Antibody rates according to symptoms.

			counts	min	25 perc	50 perc	75 perc	max	mean	St. dev	*p* value^a^
Class symptoms phase 1 (subjects with IgG ≥12)	Asymptomatic		91	−1.5	−0.13	−0.05	0.09	2.39	0.04	0.5	0.00003
	Paucisymptomatic		203	−3.39	−0.08	0.02	0.21	4.51	0.14	0.56	0.32865
	Symptomatic		319	−2.49	−0.06	0.07	0.28	16.32	0.24	1.16	0.00057
Symptoms phase 1 (subjects with IgG ≥12)	Fever	No	350	−3.39	−0.09	0.01	0.21	4.51	0.13	0.02725	0.02725
		Yes	263	−2.49	−0.06	0.05	0.25	16.32	0.24		
	Low-grade fever	No	481	−3.39	−0.08	0.02	0.23	16.32	0.19	0.17265	0.17265
		Yes	132	−0.39	−0.07	0.05	0.26	2.55	0.14		
	Cough	No	372	−3.39	−0.08	0.01	0.19	16.32	0.14	0.01120	0.01120
		Yes	241	−2.49	−0.07	0.07	0.31	10.37	0.24		
	Sore throath	No	353	−3.39	−0.09	0.02	0.23	16.32	0.19	0.08309	0.08309
		Yes	260	−2.49	−0.07	0.04	0.25	4.51	0.16		
	Muscle pain	No	299	−1.5	−0.1	0	0.2	4.51	0.13	0.00763	0.00763
		Yes	314	−3.39	−0.06	0.06	0.27	16.32	0.22		
	Asthenia	No	341	−3.39	−0.1	0	0.21	16.32	0.17	0.00574	0.00574
		Yes	272	−2.49	−0.06	0.07	0.25	10.37	0.19		
	Anosmia / dysgeusia	No	313	−3.39	−0.12	−0.01	0.2	16.32	0.14	0.00006	0.00006
		Yes	300	−0.86	−0.05	0.06	0.28	10.37	0.22		
	Gastrointestinal symptoms	No	403	−3.39	−0.08	0.03	0.21	10.37	0.17	0.46477	0.46477
		Yes	210	−2.49	−0.08	0.02	0.25	16.32	0.19		
	Conjunctivitis	No	517	−3.39	−0.08	0.02	0.21	16.32	0.18	0.16050	0.16050
		Yes	96	−2.49	−0.07	0.04	0.32	1.72	0.14		
	Dyspnea	No	493	−3.39	−0.08	0.02	0.23	16.32	0.16	0.34700	0.34700
		Yes	120	−2.49	−0.07	0.05	0.26	10.37	0.24		
	Chest pain	No	502	−3.39	−0.08	0.01	0.24	10.37	0.15	0.08088	0.08088
		Yes	111	−0.39	−0.04	0.07	0.22	16.32	0.3		
	Tachycardia	No	512	−3.39	−0.08	0.01	0.21	4.51	0.13	0.02353	0.02353
		Yes	101	−2.49	−0.06	0.08	0.32	16.32	0.4		
	Pneumonia	No	568	−3.39	−0.08	0.02	0.22	16.32	0.15	0.18692	0.18692
		Yes	45	−0.88	−0.06	0.06	0.38	10.37	0.51	1.69	

^a^Wilcoxon–Mann–Whitney test.

We analyzed the distribution of the rate feature and found a high value of kurtosis (461) around the median value of 0.016, hence to perform a multivariate analysis we restricted the data set to subjects with IgG rates either below the 10th percentile or above the 90th percentile to prevent a bias-variance problem in machine learning models and subjected the data to a linear regression analysis between the training and test data sets, where the target variable (rate of antibodies) was standardized using the Yeo–Johnson method^12^. We then applied Chi-squared statistical test to evaluate differences between classes and the rate thresholds described above (Tables 3 and 4). In order to evaluate the possible interactions between features and the rate of antibody response, we developed a multivariate approach to perform a binary classification between subjects who increased or decreased the level of antibodies. A set of seven logistic regressions has been applied on data using a bootstrap procedure (samples are drawn with replacement) and the output of each classifier has been averaged by a Bagging classifier to obtain the final output. The selection of hyperparameters of the machine learning model and the feature selection has been performed with a Bayesian optimization approach based on cross validation (fourfolds, stratified by outcome). The comparisons shown in Fig. 2 and Supplementary Fig. 2 were carried out using a one-tailed Wilcoxon matched-pairs signed-rank test. A probability value of *P* < 0.05 was considered significant. Data analyses were carried out using GraphPad Prism version 8 and Python version 3.8 with the following libraries: Pandas (version 1.1.4, data wrangling), Scipy (version 1.3.2, statistical analysis), Scikit-Learn (version 0.24.1, LR statistical model).

**Table 3:** Chi-squared analysis of groups <10th percentile and >90th percentile.

		<10 perc	>90 perc	*p* value
Sex	F	321	340	0.0627
	M	133	105	
Age	21–30	93	83	0.5429
	31–40	108	94	0.3804
	41–50	121	142	0.0970
	51–60	88	98	0.3711
	60+	44	28	0.0793
BMI^a^	18.5≤ BMI < 25	267	255	0.6963
	BMI ≥ 30	58	72	0.1750
	25≤ BMI < 30	106	93	0.4214
	BMI < 18.5	23	25	0.8261
Role	Other^b^	63	37	0.0109
	Anesthesiologist	6	7	0.9701
	Biologist	5	2	0.4640
	Surgeon	25	21	0.7007
	Physiotherapist	6	7	0.9710
	Nurse	113	132	0.1255
	Physician	56	61	0.6082
	Healthcare Partner Operator	38	64	0.0062
	Front office (PARC)	31	28	0.8494
	Researcher	13	9	0.5485
	Cleaning service	7	9	0.7698
	Transport Service	5	5	0.7747^c^
	Staff	69	44	0.0214
	Student	5	4	0.9759^c^
	Laboratory Technician	6	7	0.9701
	Radiology Technician	6	8	0.7587
Site	Other^b^	6	3	0.5223^c^
	Casa di Cura Cellini	19	8	0.0573
	Clinica Fornaca di Sessant	18	12	0.3828
	Humanitas Castelli	23	47	0.0032
	Humanitas Gavazzeni	97	142	0.0005
	Humanitas Gradenigo	36	22	0.0918
	Humanitas Mater Domini	20	23	0.7041
	Humanitas Medical Care	8	3	0.2379^c^
	Humanitas Rozzano	190	160	0.0806
	Humanitas San Pio X	27	21	0.5025
	Humanitas University	10	4	0.1905^c^

^a^Some subjects did not indicate their BMI.

^b^Refers to volunteers and other professionals that operate in several structures.

^c^Minority class is less or equal to 5 (chi-square test is not reliable).

**Table 4:** Chi-squared analysis of groups <10th percentile and >90th percentile per symptoms.

		<10 perc	>90 perc	*p* value	% Yes <10 perc^a^	%Yes >90 perc^a^
Symptoms						
Fever	No	360	284	3.9E-07		
	Yes	94	161		37	63
Low-grade fever	No	388	359	0.0678		
	Yes	66	86		43	57
Cough	No	335	284	0.0016		
	Yes	119	161		43	58
Sore throat	No	302	271	0.0923		
	Yes	152	174		47	53
Muscle pain	No	310	246	0.0001		
	Yes	144	199		42	58
Asthenia	No	340	268	3.7E-06		
	Yes	114	177		39	61
Anosmia/dysgeusia	No	364	247	4.0E-15		
	Yes	90	198		31	69
Gastrointestinal symptoms	No	336	313	0.2485		
	Yes	118	132		47	53
Conjunctivitis	No	400	382	0.3633		
	Yes	54	63		46	54
Dyspnea	No	405	375	0.0370		
	Yes	49	70		41	59
Chest pain	No	415	367	0.0001		
	Yes	39	78		33	67
Tachycardia	No	406	371	0.0107		
	Yes	48	74		39	61
Pneumonia	No	440	420	0.0889		
	Yes	14	25		36	64
Total of Symptoms in phase 1	Asymptomatic	150	80	3.4E-07	65	35
	Paucisymptomatic	179	158	0.2520	53	47
	Symptomatic	125	207	5.6E-09	38	62
Comorbidities						
Chronic obstructive bronchopneumopathy	No	451	444	0.6305^b^		
	Yes	3	1			
Asthma	No	430	412	0.2408		
	Yes	24	33			
Dyslipidemia	No	413	404	0.9834		
	Yes	41	41			
Past neoplasia	No	431	438	0.0063		
	Yes	23	7			
Hypertension	No	401	409	0.0915		
	Yes	53	36			
Past coronaropathies	No	454	443	0.4702^b^		
	Yes	0	2			
Atrial fibrillation	No	450	441	0.7439^b^		
	Yes	4	4			
Past stroke/ TIA	No	452	445	0.4878^b^		
	Yes	2	0			
Steatosis/cyrrosis	No	448	444	0.1359^b^		
	Yes	6	1			
Chronic kidney failure	No	453	445	0.9920^b^		
	Yes	1	0			
Other liver diseases	No	452	441	0.6641^b^		
	Yes	2	4			
Rheumatological diseases	No	445	431	0.3715		
	Yes	9	14			
Other diseases of the immune system	No	418	409	0.9726		
	Yes	36	36			
Diabetes mellitus	No	452	443	0.6305^b^		
	Yes	2	2			
Gotta	No	452	445	0.4878^b^		
	Yes	2	0			

^a^Percentage of subjects with symptoms (Yes) per rate class.

^b^Minority class is less or equal to 5 (chi-square test is not reliable).

### Reporting summary

Further information on research design is available in the Nature Research Reporting Summary linked to this article.

## Results

### Rate of the antibody response during phase 1 and phase 2 of SARS-CoV-2 diffusion

We analyzed the persistence of the antibody response in healthcare workers that underwent immunological surveillance for SARS-CoV-2 exposure and resulted positive for anti-Spike 1/2 IgG (IgG ≥12 AU/mL). Although the test manufacturer considers positive subjects above 15 AU/mL and equivocal those between 12 and 15 (AU/mL), based on our previous publication showing that these two groups behaved very similarly we considered positive everybody ≥12 AU/mL^10^. The accrual was on a voluntary basis and did not occur in the symptomatic phase (at around 43 ± 17 days from COVID-19 assessment when symptomatic) (*n* = 4735). We excluded all of the individuals that became positive over the course of the analysis (*n* *=* 2) and those that dropped from phase 1 or for which we were missing at least two features, so to focus only on those individuals that were exposed during the first wave of infection to evaluate the duration of the antibody response (*n* = 4534). We assessed the correlation of the rate of antibody increase or decrease with the different analyzed features for the first two time points of observation in subjects with IgG ≥12 AU/mL. In Tables 1 and 2 are reported the rates for individual classes of features with relative statistical analysis. As shown, females sustained the antibody response better than males (*p* = 0.01); similarly, nonmedical healthcare professionals (specifically, healthcare partner operators) had higher antibody rates (*p* = 0.0009). The levels of antibodies increased in hospitals located in the Bergamo area (Castelli and Gavazzeni *p* < 0.0001) (Table 1) which was more hit by COVID-19 (37–43% of individuals with IgG ≥12)^10^. More important, the IgG rate in individuals which were positive for IgG (IgG ≥12 AU/mL; *n* = 613) at the beginning of the study was increased (*p* < 0.000001) over time, and this increase was either minor in asymptomatics (*n* = 91, *p* = 0.00003) and paucisymptomatics (*n* = 203) or strong in symptomatics (*n* = 319, *p* = 0.0006) (Table 2). This may explain why individuals from hospitals in the Bergamo area or nonmedical healthcare professionals had higher levels of antibodies as most of them suffered from symptomatic COVID-19 (59 and 73%, respectively). On the contrary, those that had an intermediate IgG titer (3.8 < IgG <12 AU/mL considered as negative) displayed a significant reduction in IgG rate (*p* < 0.000001) (Table 1). However, this population is considered as negative for SARS-CoV-2 IgG according to the manufacturer. Many symptoms, including fever, cough, muscle pain, asthenia, tachycardia, and anosmia/dysgeusia, correlated with an increase of antibodies in the first two time points of observation (Table 2).

### The rate of the antibody response depends on the symptoms

**Fig. 1:**
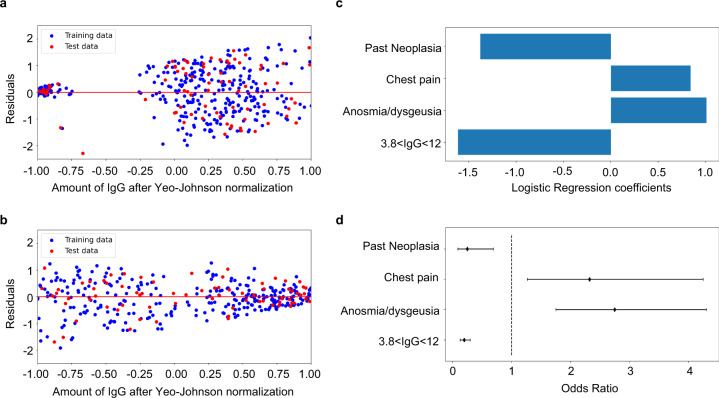
Results of linear and logistic regression for rate analysis in patients with >90th percentile and <10th percentile. **a** Dataset <10th percentile regression diagnostic plot of amount of IgG after Yeo-Johnson normalization against residuals of training and test data (*n* *=* 454); **b** Dataset >90th percentile regression diagnostic plot of amount of IgG after Yeo-Johnson normalization against residuals of training and test data (*n* *=* 445); **c** Barplot with logistic regression coefficients for most important features; **d** Odds ratio of logistic regression with confidence intervals (95%) for the most important features.

As we noticed that the distribution of the rate feature presented a high value of kurtosis (see Methods) we restricted the data set to subjects with IgG rates either below the 10th percentile [<−0.033 (*n* = 454)] or above the 90th percentile [>0.005 (*n* = 445)] to prevent a bias-variance problem in machine learning models. The accuracy of these rates was confirmed by linear regression analysis. In Fig. 1a, b are shown the regression diagnostic plots of predicted values against residuals of training and test data according to the threshold (<−0.033 AU/mL*day and >0.005 AU/mL*day). In Table 3 is shown the Chi-squared analysis for the populations below or above the set threshold rates. We found that as for the previous analysis, males reduced the level of antibodies more than females, even though this difference was no longer statistically significant (*p* = 0.06). The levels of antibodies increased in hospitals located in the Bergamo area (Castelli and Gavazzeni: *p* = 0.0032 and *p* = 0.0005, respectively) which was more hit by COVID-19 (37–43% of individuals with IgG ≥12) and most of the individuals were symptomatic^10^ while it decreased in Humanitas Rozzano (*p* = 0.0806) which was less heavily hit (10% of individuals with IgG ≥12) and had less symptomatic individuals^10^ (Table 3). The rate decreased in asymptomatic (65% of subjects fell in the group <−0.033; *p* < 0.000001), remained constant in paucisymptomatic, and increased in symptomatic individuals (62% of subjects were in the group >0.005; *p* < 0.000001) (Table 4). Interestingly, among the different symptoms, fever, cough, muscle pain, asthenia, dyspnea, tachycardia, chest pain, and anosmia/dysgeusia all correlated with a higher number of individuals falling into the group with rate >0.005, indicating that these symptoms were strongly associated with sustained/increased antibody response (0.000001 < *p* < 0.05, Table 4 and Supplementary Fig. 1). Among these, anosmia/dysgeusia was associated with the highest percentage of subjects presenting with increased IgG rate (69%; *p* < 0.000001, Table 4 and Supplementary Fig. 1).

### Increase of antibody response in subjects with anosmia/dysgeusia or chest pain

Having observed differences according to sex, role, and site, and since many symptoms are linked, we performed a multivariate statistical analysis based on a supervised machine learning classification approach (see Methods). Through a Bayesian hyperparameter optimization algorithm, we assessed that the best machine learning model is a Bagging classifier of seven logistic regression, which was evaluated both on a training (Accuracy = 76.26; ROC AUC = 76.30; Recall = 81.14) and a test dataset (Accuracy = 72.00; ROC AUC = 72.12; Recall = 81.08), where the training/test split is 80–20%, stratified by the outcome. Classification metrics on the training set and test set are comparable, which shows that the model does not present overfitting on training data. In Fig. 1c is shown the multivariate logistic regression analysis. We found that the increased rate was associated primarily with anosmia/dysgeusia (regression coefficient *=* 1.0, 95% CI 0.56–1.46) and with chest pain (regression coefficient *=* 0.84, 95% CI 0.24–1.44), while the decreased rate was associated to subjects with intermediate IgG (3.8 < IgG <12) (regression coefficient = −1.61, 95% CI −2.03 to −1.0), which may be related to a noise in the instrument testing, and with past neoplasia (regression coefficient = −1.38, 95% CI −2.4 to −0.37). Interestingly, 54% of subjects with chest pain also presented loss of smell/taste while only 22% of subjects with smell/taste dysfunction also had chest pain, suggesting that IgG increase in the symptomatic population is primarily linked to anosmia and dysgeusia (not shown). In Fig. 1d are shown the odds ratio relative to Fig. 1c, which for chest pain is 2.32 (95% CI 1.27–4.24), for anosmia/dysgeusia is 2.75 (95% CI 1.75–4.30), for subjects with intermediate IgG (3.8 < IgG <12) is 0.2 (95% CI 0.13–0.30), and for subjects with past neoplasia is 0.25 (95% CI 0.09–0.69). Overall, these results indicate that although many symptoms are associated with an increase of IgG abundance in the observation time, only anosmia/dysgeusia and chest pain are associated to a higher IgG rate in the multivariate logistic regression analysis. By contrast the population with past neoplasia or intermediate levels of IgG (3.8 < IgG <12 AU/mL) are the ones that display a reduction in IgG. However, the significance of the reduction in the detection of antibodies in subjects with intermediate levels of IgG remains to be investigated, as this population did not represent early infected individuals because they were all nasopharyngeal swab negative^10^.

### The antibody response lasts for at least 8–10 months

**Fig. 2:**
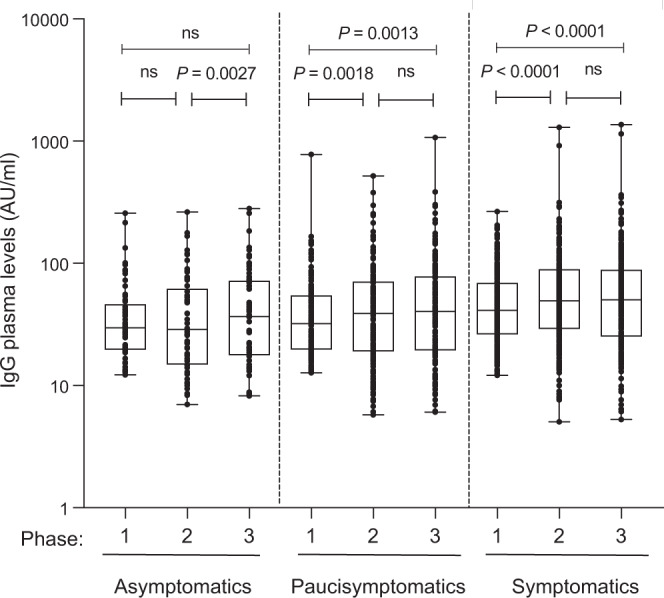
Anti-Spike S1/S2 IgG plasma levels. Anti-Spike S1/S2 IgG plasma levels were measured in asymptomatics (*n* *=* 61), paucisymptomatics (*n* *=* 163), and symptomatics (*n* *=* 275) at three different time points (phase 1–3). Each dot corresponds to an individual subject. Log scale on the Y axis. The box plots show the interquartile range, the horizontal lines show the median values, and the whiskers indicate the minimum-to-maximum range. *P* values were determined using a one-tailed Wilcoxon matched-pairs signed rank test.

We then analyzed whether the antibody response was maintained over time in the third time point of analysis (*n* = 499) which was evaluated between November and December 2020 thus reaching an observation of 8–10 months. As shown in Fig. 2 and Supplementary Fig. 2 we observed that both symptomatic and paucisymptomatic individuals still displayed a higher level of antibodies, however, they did not increase between phase 2 and phase 3. By contrast, asymptomatic individuals did not increase their IgG levels over time.

## Discussion

We analyzed the 8–10-month duration of an antibody response to SARS-CoV-2 in personnel from nine healthcare facilities and an international medical school (Humanitas University) in Northern Italy in areas differently hit by the virus^10^. We show that the antibody response is stable both in symptomatic and asymptomatic/paucisymptomatic individuals and is increased in females and in nonmedical healthcare professionals. Previously, it has been shown in a study conducted in the British population that the antibody response declines of nearly 22% in symptomatic individuals and of 64% in asymptomatic individuals^2^. However, this study was based on a prick qualitative test and thus the decline may be related to the sensitivity of the test. We also observed that the antibody response declined when we analyzed the group (3.8 < IgG < 12 AU/mL) with IgG between the limit of detection (3.8 AU/mL) and the threshold of positivity (IgG ≥12 AU/mL), as set by the manufacturer. Whether this is linked to a difference linked to the sensitivity of the test or to a real reduction in an antibody response that may or may not be specific to SARS-CoV-2, remains to be established. In a previous analysis, we excluded that this population represented individuals in the initial phases of an infection as all of them were tested for SARS-CoV-2 by a nasopharyngeal swab which however resulted negative^10^. When we analyzed the extremes, i.e., the individuals with higher rates of antibody increase or decrease (<−0.033 and >0.005 AU/mL*day) we observed that asymptomatics had higher negative rates while symptomatics tended to continue increasing the antibody levels suggesting that extreme changes in rate separate the symptomatics from the asymptomatics. As during the observation time there was very limited viral diffusion in Northern Italy, as confirmed also by the finding that only two individuals became IgG positive and 2981 remained IgG negative throughout the study (all excluded from the analysis), we can conclude that the sustained or augmented antibody response may not be linked to reexposure to the virus.

In an attempt to address what improved the antibody response, we found that several symptoms were associated with increased rates of antibodies, however, in the multivariate logistic analysis, only anosmia/dysgeusia and chest pain were linked with the highest regression coefficients. Chest pain and anosmia are long-lasting symptoms in COVID-19 patients^13^. In addition, anosmia and/or dysgeusia are very common as they are found in around 50–70% of subjects affected by COVID-19^14,15^. In our cohort (Table 2), 49% of IgG-positive subjects had anosmia/dysgeusia, 28% chest pain, and 13.7% both anosmia/dysgeusia and chest pain, suggesting that indeed these two symptoms may, either alone or in combination, associate with IgG increase. We and others previously found that anosmia/dysgeusia together with fever were the symptoms that mostly characterized SARS-CoV-2 exposure^10,16^. In agreement, anosmia and dysgeusia have been proposed to be used to track SARS-CoV-2 diffusion^17^. Interestingly, SARS-CoV-2 can infect the olfactory epithelium^18,19^, including olfactory sensory neurons, support cells, and immune cells, that express the viral entry receptors ACE2 and TMPRSS2^18,20,21^. Here, the virus can persist long and induce local inflammation^19^ and olfactory bulb abnormalities^22–24^. In agreement, the loss of smell and taste can persist in individuals even with RT-PCR SARS-CoV-2 negativity in the nasopharyngeal swab for months^19,25^. Supporting this possibility, we did not detect any further increase of IgG levels between phase 2 and phase 3 suggesting that when individuals eliminate the virus then there is no further increase of the antibody response.

One limitation of our study is that we followed our healthcare workers for the exposure to SARS-CoV-2 via measuring the anti S1/S2 IgG response and have not evaluated any other antibody subtype nor their neutralizing activity, even though the test used has correlated the antibody levels with their neutralizing activity, as reported in the Methods section.

Overall, these data suggest that increased antibody response in patients with anosmia/dysgeusia may be linked to the persistence of the virus in the olfactory bulb which through local inflammation and release of antigens, maintains and boosts the antibody response. This study opens new perspectives on the immunity to SARS-CoV-2 and warrants further investigation on the role of anosmia/dysgeusia on antibody response through the design of prospective observational studies coupling the testing of SARS-CoV-2 persistence in the olfactory bulb, loss of smell or taste and antibody titers. In addition, we show that the antibody response to the natural infection is durable and persists for at least 8 months. If the antibody response elicited by the vaccines is similarly effective, we may expect it to last for at least the same amount of time. Further, this observation strongly supports our findings and those of others that convalescent symptomatic COVID-19 patients should receive only one dose of vaccine^26–29^ and suggests that this may occur even at months of distance from developing the disease as their antibody response will just need to be boosted.

## Supplementary information

Supplementary Information

Supplementary Data 1

Description of Additional Supplementary Files

Peer Review File

Reporting Summary

## Data Availability

The dataset is available at the link https://zenodo.org/record/5266408^30^ with restricted license however available upon request. Patient informed consent does not allow for the deposition of clinical data in public access repositories. Interested researchers should contact biblioteca@humanitas.it to inquire about access; requests for noncommercial academic use will be considered and require ethics review. Source data for the main figures in the manuscript can be accessed as Supplementary Data 1.

## Code availability

Code developed for the analysis can be accessed at the link https://zenodo.org/record/5266426#.YSdIXN_ONaQ^31^.
