# Supplementary material for: The antibody response to SARS-CoV-2 infection persists over at least 8 months in symptomatic patients
Source: Commun Med (Lond). 2021 Sep 17;1:32. doi: 10.1038/s43856-021-00032-0 (PMC8767777; doi:10.1038/s43856-021-00032-0)
Supplement: Supplementary file 1 — Supplementary Information [file 43856_2021_32_MOESM1_ESM.pdf]

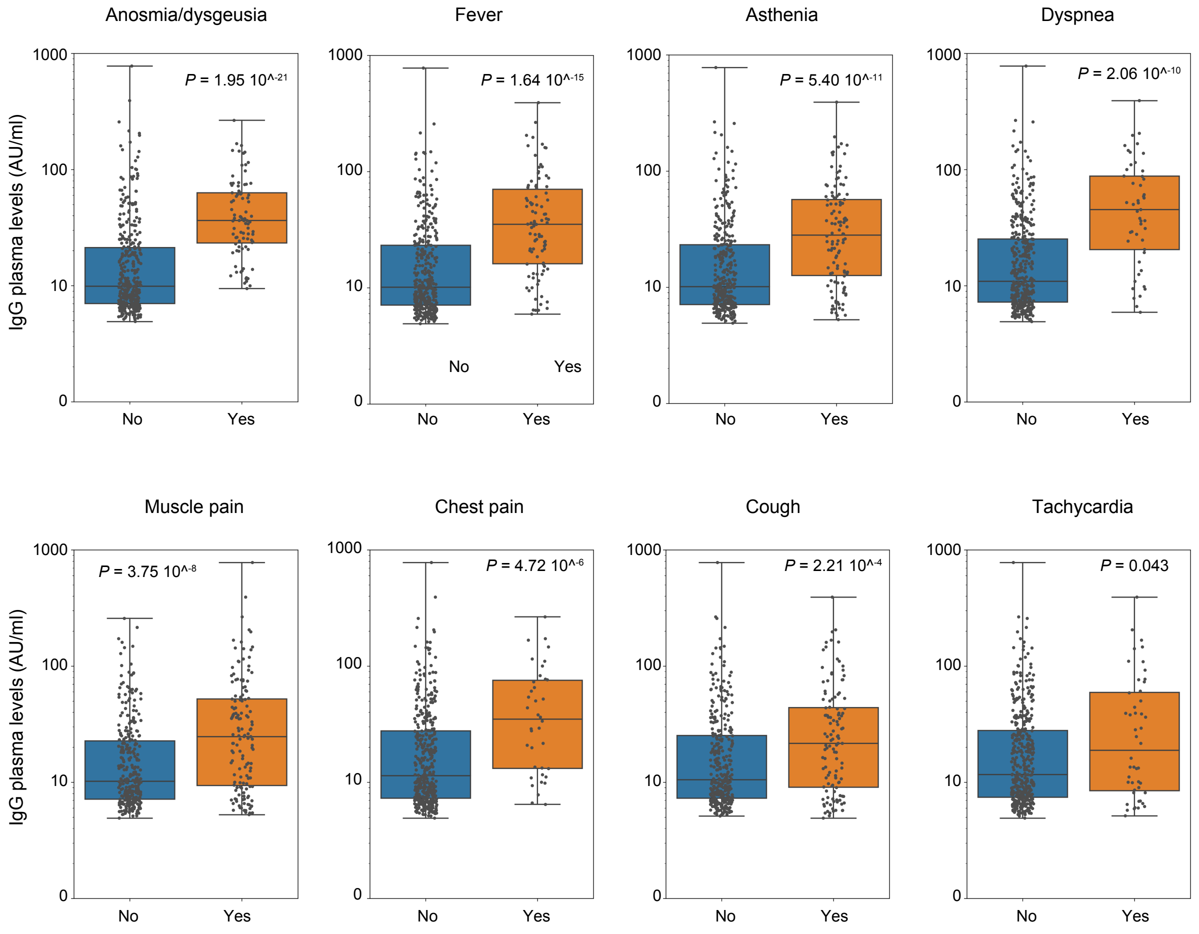

Supplementary Figure 1. Anti-Spike S1/S2 IgG plasma levels and symptoms. Box plots showing Anti-Spike S1/S2 IgG plasma levels for statistically significant symptoms in patients included in the regression model, with rates of antibodies >90th percentile (n=445) and <10th percentile (n=454). The box plots show the interquartile range, the horizontal lines show the median values and the whiskers indicate the minimum-to- maximum range. *P*-values were determined using two-tailed Mann-Whitney U rank test for binary comparison.

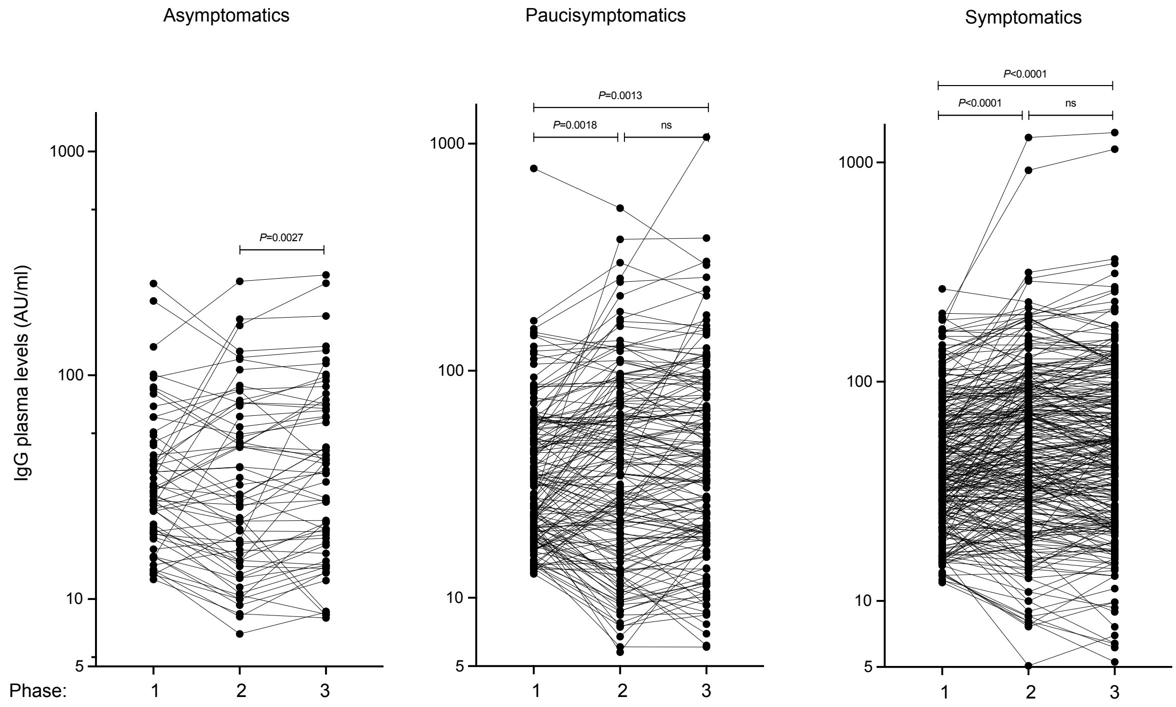

Supplementary Figure 2. Anti-Spike S1/S2 IgG plasma levels. Anti-Spike S1/S2 IgG plasma levels in asymptomatics (n=61), pauci-symptomatics (n=163) and symptomatics (n=275) measured at three different time points (phase 1-3). Connecting lines of dots correspond to the same individual in three different time points. Log scale on Y axis. *P*-values were determined using one-tailed Wilcoxon matched-pairs signed rank test.

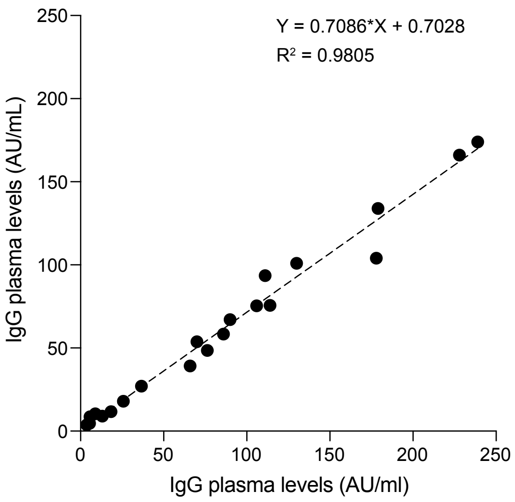

Supplementary Figure 3. Accuracy test on samples from the phase 1 (n=21) with the detection kits of phase 1 (x axis) and phase 2 (y axis).
