# Supplementary material for: The antibody response to SARS-CoV-2 infection persists over at least 8 months in symptomatic patients
Source: Commun Med (Lond). 2021 Sep 17;1:32. doi: 10.1038/s43856-021-00032-0 (PMC8767777; doi:10.1038/s43856-021-00032-0)
Supplement: Supplementary file 3 — Description of Additional Supplementary Files [file 43856_2021_32_MOESM3_ESM.pdf]

Description of Additional Supplementary Files

File Name: Supplementary Data 1

Description: Source data for the main figures 1 and 2.
