# Supplementary material for: The antibody response to SARS-CoV-2 infection persists over at least 8 months in symptomatic patients
Source: Commun Med (Lond). 2021 Sep 17;1:32. doi: 10.1038/s43856-021-00032-0 (PMC8767777; doi:10.1038/s43856-021-00032-0)
Supplement: Supplementary file 5 — Reporting Summary [file 43856_2021_32_MOESM5_ESM.pdf]

nature research

Corresponding author(s): Prof. Maria Rescigno, maria.rescigno@hunimed.eu

Last updated by author(s): Aug 27, 2021

Reporting Summary

Nature Research wishes to improve the reproducibility of the work that we publish. This form provides structure for consistency and transparency in reporting. For further information on Nature Research policies, see our Editorial Policies and the Editorial Policy Checklist.

Statistics

For all statistical analyses, confirm that the following items are present in the figure legend, table legend, main text, or Methods section.

n/a	Confirmed	
☐	☒	The exact sample size (n) for each experimental group/condition, given as a discrete number and unit of measurement
☐	☒	A statement on whether measurements were taken from distinct samples or whether the same sample was measured repeatedly
☐	☒	The statistical test(s) used AND whether they are one- or two-sided<br>*Only common tests should be described solely by name; describe more complex techniques in the Methods section.*
☐	☒	A description of all covariates tested
☐	☒	A description of any assumptions or corrections, such as tests of normality and adjustment for multiple comparisons
☐	☒	A full description of the statistical parameters including central tendency (e.g. means) or other basic estimates (e.g. regression coefficient) AND variation (e.g. standard deviation) or associated estimates of uncertainty (e.g. confidence intervals)
☐	☒	For null hypothesis testing, the test statistic (e.g. F, t, r) with confidence intervals, effect sizes, degrees of freedom and P value noted<br>*Give P values as exact values whenever suitable.*
☒	☐	For Bayesian analysis, information on the choice of priors and Markov chain Monte Carlo settings
☒	☐	For hierarchical and complex designs, identification of the appropriate level for tests and full reporting of outcomes
☒	☐	Estimates of effect sizes (e.g. Cohen's d, Pearson's r), indicating how they were calculated

Our web collection on statistics for biologists contains articles on many of the points above.

Software and code

Policy information about availability of computer code

Data collection

This observational study has been approved by the institutional review board of Istituto Clinico Humanitas for all participating institutes (ID:1374 IgG COVID-19 Humanitas and registered on clinicaltrial.gov NCT04387929). Accrual was on a voluntary basis: it started on April 28th and more than 80% of personnel participated (n = 4735). The study foresees 4 blood collections every 3/4 months. All participants signed an informed consent and filled a questionnaire before blood collection. We analyzed 93 features (72 categorical and 17 numerical and 4 temporal) including, age, sex, location, professional role, time between sample collections, COVID-19 symptoms (fever, sore throat, cough, muscle pain, asthenia, anosmia/dysgeusia (loss of smell and taste), gastrointestinal symptoms, conjunctivitis, dyspnea, chest pain, tachycardia, pneumonia), home exits and smart-working, comorbidities (diabetes, asthma, neoplasia, autoimmunity, cardiovascular disorders, hepatic disorders).We considered “asymptomatics” subjects without any symptoms; “paucisymptomatics” individuals that developed 1 or 2 symptoms; “symptomatics” individuals with more than 3 symptoms. None of the participants were enrolled at the time of symptoms. Thus, when the first serological test was performed, they were either asymptomatics or the symptoms had disappeared. After excluding for employees that became positive for SARS-CoV-2 IgG (n = 2) during the observation period and those that dropped from phase 1 or for which we were missing at least two features, we analyzed 4534 participants (4.25% drop out) that participated to both phase 1 (April-May 2020) and phase 2 (July-August 2020). Among these, 499 subjects participated also to phase 3 (November-December 2020).

Code developed for the analysis can be accessed at the link https://zenodo.org/record/5266426#.YSdIXN_ONaQ.

Data analysis

Analysis were developed in open source environment Python 3.7 using data science packages including pandas 0.25.3 (data wrangling), NumPy 1.17.5 (computations), SciPy 1.4.1 (hypothesis testing), and Scikit-learn 0.22.1 (modelling).

For manuscripts utilizing custom algorithms or software that are central to the research but not yet described in published literature, software must be made available to editors and reviewers. We strongly encourage code deposition in a community repository (e.g. GitHub). See the Nature Research guidelines for submitting code & software for further information.

Data

Policy information about availability of data

All manuscripts must include a data availability statement. This statement should provide the following information, where applicable:

- Accession codes, unique identifiers, or web links for publicly available datasets
- A list of figures that have associated raw data
- A description of any restrictions on data availability

The dataset is available at the link https://zenodo.org/record/5266408 with restricted license however available upon request. Patient informed consent does not allow for deposition of clinical data in public access repositories. Interested researchers should contact biblioteca@humanitas.it to inquire about access; requests for noncommercial academic use will be considered and require ethics review. Source data for the main figures in the manuscript can be accessed as Supplementary Data 1.

Field-specific reporting

Please select the one below that is the best fit for your research. If you are not sure, read the appropriate sections before making your selection.

☒ Life sciences ☐ Behavioural & social sciences ☐ Ecological, evolutionary & environmental sciences

For a reference copy of the document with all sections, see nature.com/documents/nr-reporting-summary-flat.pdf

Life sciences study design

All studies must disclose on these points even when the disclosure is negative.

Sample size	This was an observational study aimed at determining the anti-SARS-CoV-2 IgG plasma levels in nearly 4000 employees (n = 4735) of 9 healthcare facilities and an international medical school located in northern Italy.<br>The serological determination was offered to all the employees of the involved sites and the anticipated refusal rate was assumed to be 10-15%. The overall IgG positivity was assumed to be around 10-15%.<br>Primary endpoint was the number of test positive subjects. Given the study size, the study was able to estimate the overall positivity with a width of 95% confidence interval equal to 2% and the positivity for subgroups of at least 200 patients with a width of 95% confidence interval equal to 9%. No power analysis was performed to calculate the sample size.
Data exclusions	After excluding for employees that became positive for SARS-CoV-2 IgG (n = 2) during the observation period and those that dropped from phase 1 or for which we were missing at least two features, we analyzed 4534 participants (4.25% drop out). Among these, 499 subjects participated also to phase 3.
Replication	n.a.
Randomization	No randomisation was performed.
Blinding	n.a.

Reporting for specific materials, systems and methods

We require information from authors about some types of materials, experimental systems and methods used in many studies. Here, indicate whether each material, system or method listed is relevant to your study. If you are not sure if a list item applies to your research, read the appropriate section before selecting a response.

Materials & experimental systems

n/a	Involved in the study	
☒	☐	Antibodies
☒	☐	Eukaryotic cell lines
☒	☐	Palaeontology and archaeology
☒	☐	Animals and other organisms
☐	☒	Human research participants
☐	☒	Clinical data
☒	☐	Dual use research of concern

Methods

n/a	Involved in the study	
☒	☐	ChIP-seq
☒	☐	Flow cytometry
☒	☐	MRI-based neuroimaging

Human research participants

Policy information about studies involving human research participants

Population characteristics	This observational study aimed at determining the anti-SARS-CoV-2 IgG plasma levels in nearly 4000 employees of 9 healthcare facilities and an international medical school located in northern Italy.
Recruitment	The study has been approved by the institutional review board of Istituto Clinico Humanitas for all participating institutes (ID:1374 IgG COVID-19 Humanitas and registered on clinicaltrial.gov NCT04387929). We have adhered to the STROBE reporting guidelines for observational studies. Accrual was on a voluntary basis: it started on April 28th and more than 80% of personnel participated. 10 different centers participate: Istituto Clinico Humanitas (ICH), Rozzano (MI); Humanitas Gavazzeni, Bergamo; Humanitas Castelli, Bergamo; Humanitas Mater Domini (HMD), Castellanza (VA); Humanitas Medical Center, HMC, Varese; Humanitas University, Pieve Emanuele (MI); Humanitas San Pio X, Milano; Humanitas Cellini, Torino; Humanitas Gradenigo, Torino; Clinica Fornaca, Torino. All participants signed an informed consent and filled an anamnestic questionnaire before blood collection. In order to be tested, subjects had to fill in the questionnaire. Only after filling the questionnaire in its entirety, would the individuals be scheduled for blood sampling. Hence, we obtained a 100% complete data. None of the participants were enrolled at the time of symptoms. Thus, when the serological test was performed, they were either asymptomatics or the symptoms had disappeared.
Ethics oversight	The study was conducted following the Helsinki principles of good clinical practice and it has been approved by the institutional review board of Istituto Clinico Humanitas for all participating institutes (ID:1374 IgG COVID-19 Humanitas) and registered on clinicaltrial.gov (NCT04387929). All participants signed an informed consent.

Note that full information on the approval of the study protocol must also be provided in the manuscript.

Clinical data

Policy information about clinical studies

All manuscripts should comply with the ICMJE guidelines for publication of clinical research and a completed CONSORT checklist must be included with all submissions.

Clinical trial registration	The study has been approved by the institutional review board of Istituto Clinico Humanitas for all participating institutes (ID:1374 IgG COVID-19 Humanitas) and registered on clinicaltrial.gov (NCT04387929).
Study protocol	The full trial protocol can be accessed.
Data collection	Accrual was on a voluntary basis: it started on April 28th and more than 80% of personnel participated (n = 4735). The study foresees 4 blood collections every 3/4 months. All participants signed an informed consent and filled a questionnaire before blood collection. We analyzed 93 features (72 categorical and 17 numerical and 4 temporal) including, age, sex, location, professional role, time between sample collections, COVID-19 symptoms (fever, sore throat, cough, muscle pain, asthenia, anosmia/dysgeusia (loss of smell and taste), gastrointestinal disorders, conjunctivitis, dyspnea, chest pain, tachycardia, pneumonia), home exits and smart-working, comorbidities (diabetes, asthma, neoplasia, autoimmunity, cardiovascular disorders, hepatic disorders). We considered “asymptomatics” subjects without any symptoms; “paucisymptomatics” individuals that developed 1 or 2 symptoms; “symptomatics” individuals with more than 3 symptoms. None of the participants were enrolled at the time of symptoms. Thus, when the first serological test was performed, they were either asymptomatics or the symptoms had disappeared. After excluding for employees that became positive for SARS-CoV-2 IgG (n = 2) during the observation period and those that dropped from phase 1 or for which we were missing at least two features, we analyzed 4534 participants (4.25% drop out) that participated to both phase 1 (April-May 2020) and phase 2 (July-August 2020). Among these, 499 subjects participated also to phase 3 (November-December 2020).
Outcomes	The outcome was the measurement of the IgG plasma levels.<br>The outcome measures were analysed for their association with basic demographic characteristics (including age, sex, location, professional role), time between sample collections, COVID-19 symptoms (fever, sore throat, cough, muscle pain, asthenia, anosmia/dysgeusia (loss of smell and taste), gastrointestinal symptoms, conjunctivitis, dyspnea, chest pain, tachycardia, pneumonia), home exits and smart-working, comorbidities (diabetes, asthma, neoplasia, autoimmunity, cardiovascular disorders, hepatic disorders).
